# The Saskatchewan Caregiver Experience Study: Support Priorities of Caregivers of Older Adults

**DOI:** 10.1177/08445621241273956

**Published:** 2024-08-11

**Authors:** Steven Hall, Noelle Rohatinsky, Lorraine Holtslander, Shelley Peacock

**Affiliations:** ^1^College of Nursing, 7235 University of Saskatchewan, Health Science Building – 1A10, Box 6,107 Wiggins Road, Saskatoon, SK, S7N 5E5, Canada

**Keywords:** Caregivers, older adults, aging, lived experiences, qualitative description, support priorities

## Abstract

**Background:**

Population aging is a global phenomenon. Many older adults living with chronic conditions rely on family and friend caregivers. The growing demand for family and friend caregivers underscores the necessity for adequate and effective support services.

**Purpose:**

The Saskatchewan Caregiver Experience Study sought to gather the perspectives of caregivers of older adults and set priorities for caregiver support.

**Methods:**

An online survey with open-ended questions was employed in this qualitative descriptive study. In this manuscript, we present our findings from the survey question: “What do you think is most important for support in your caregiving role? In other words, what are your top priorities for support?”

**Findings:**

This survey question received n = 352 responses, evenly distributed across Saskatchewan in urban-large (33%), urban-small/medium (32%), and rural (35%) settings. Support priorities of Saskatchewan caregivers were found to be access to help when they need it; an ear to listen and a shoulder to lean on; assistance in optimizing the care recipient's health; having healthcare professionals that care; and improved policies, legislations, and regulations.

**Conclusion:**

Services and interventions that assist caregivers are more likely to be accessed and utilized when caregivers are given the opportunity to identify their own support priorities. This study has the potential to inform health and governmental systems to support caregivers of older adults provincially within Saskatchewan, nationally in Canada, and in a global context.

## Introduction

The growing population of older adults, many of whom rely on informal caregivers, presents a significant public health challenge. Caregivers are indispensable in managing chronic illnesses and providing long-term care, yet they often lack adequate support and recognition (Hall et al., 2022; Kontrimiene et al., 2021). Their experiences are characterized by a spectrum of emotional, physical, and social impacts, which can lead to burnout and decreased well-being (Hall et al., 2024a; Mayo et al., 2020). As such, it is clear that caregivers of older adults require support (Hall et al., 2022). In this paper, we identify the primary support priorities of caregivers in Saskatchewan, Canada, that were derived from the Saskatchewan Caregiver Experience Study. This information is vital for developing effective strategies and policies that address the real-world complexities of caregiving, ultimately enhancing the quality of life for both caregivers and the older adults they support.

The growing demand for family and friend caregivers underscores the necessity for adequate support services. Caregivers often face a multitude of challenges in their caregiving role (Hall et al., 2024a). Recent studies have shed light on the priorities and needs of caregivers, providing valuable insights for developing targeted support services (Hall et al., 2022). Moscou-Jackson et al. (2019) discuss the significance of interventions that support aging in place, highlighting the important role of family and friend caregivers in enabling older adults to remain in their homes. Their study identifies the need for research on patient-centered approaches that align with the needs and goals of both patients and caregivers (Moscou-Jackson et al., 2019). A 2021 Consensus Conference on Engaging Family Caregivers of Persons with Dementia emphasized the need for multidisciplinary collaborations in areas such as identification and assessment of dementia caregivers, reimbursement and financing for caregiver support, caregiver training across the care continuum, healthcare provider education on family-centered care, and technology innovations to support dementia caregivers (Riffin, 2022).

The Saskatchewan Caregiver Experience Study (Hall, 2023) was an initiative addressing a critical aspect of healthcare and social support: the experiences and needs of caregivers to older adults. With an aging population and a significant proportion of older adults living with chronic conditions, the role of family and friend caregivers becomes increasingly vital (Alzheimer's Disease International, 2021; Schulz et al., 2020). It is important to create accessible and appropriate services that are tailored to the unique needs of caregivers (Hall et al., 2022). Our study aimed to bridge this gap by understanding caregivers’ perspectives and setting priorities for support services. By focusing on caregivers’ lived experiences and identifying their primary concerns, the study seeks to inform the development of interventions and policies that are both practical and effective in meeting the complex needs of caregivers. This approach ensures that the support services are not only theoretically sound, but also resonate with the actual demands and challenges faced by caregivers in their day-to-day lives. Overall, support services and interventions for caregivers should seek to ensure the well-being and sustainability of the caregiving role.

## Materials and methods

The Saskatchewan Caregiver Experience Study (Hall, 2023) was a qualitative descriptive study conducted in the province of Saskatchewan, Canada. The methods employed in this study are reported in detail within the Hall et al. (2024b) research methodology paper, which follows the Standards for Reporting Qualitative Research (SRQR) guidelines (O’Brien et al., 2014). The research team developed an online qualitative survey, drawing insights from the qualitative survey literature by Braun et al. (2020). The survey was hosted on SurveyMonkey (Momentive Inc., 2021) and distributed via Facebook and community newsletters. To gather a diverse range of perspectives, purposive maximum variation sampling methods were used to recruit Saskatchewan caregivers who currently or previously cared for older adults at any level of involvement, aged >55 years for this study. On Facebook, the lead author (SH) joined over 70 community Facebook groups across Saskatchewan and manually shared a link to the online survey. Additionally, with a small fund allocated to our study by the Saskatoon Council on Aging, we were able to run a paid advertisement campaign on Facebook. The Saskatoon Council on Aging and the Canadian Red Cross (Saskatchewan branches) also distributed the survey link in their community newsletters.

The study received ethical approval from the University of Saskatchewan's Behavioural Research Ethics Board (Beh ID #3377). When prospective participants accessed the distributed survey link, the first page presented the consent form to the study. Participants had the opportunity to review the consent form, were provided with the researcher's contact information to seek further clarification if needed, and were informed that by clicking a “Continue to Survey” button, they were providing informed consent (Hall et al., 2024b). The survey then collected demographics and featured four open-ended qualitative questions addressing: (1) the challenges experienced by caregivers (Hall et al., 2024a); (2) the perceived positive aspects of caregiving (Hall et al., 2024c); (3) the support priorities of caregivers; and a fourth question which gave caregivers an opportunity to share additional thoughts. This manuscript reports on the findings from the third survey question regarding support priorities.

**Figure A1.**
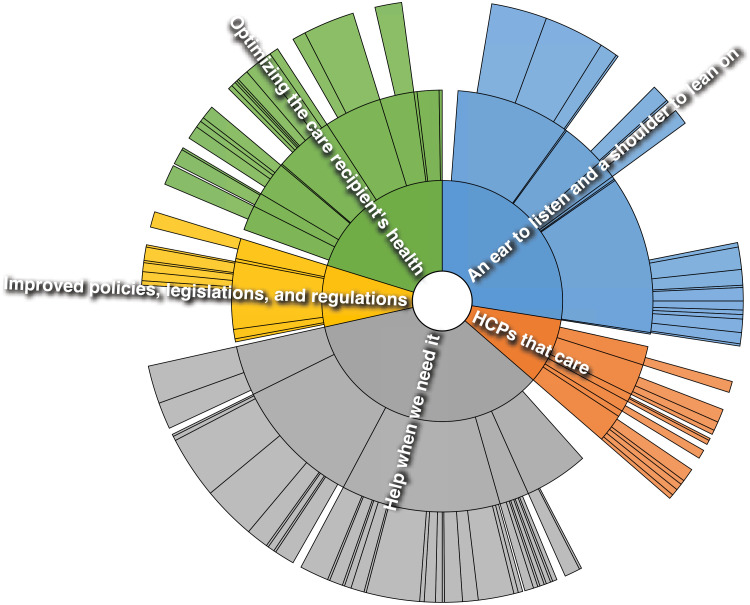
Sunburst Hierarchy Chart.

Content analysis (Hsieh & Shannon, 2005) was the method used for data analysis (Hall et al., 2024b). Firstly, we thoroughly reviewed all survey responses to gain a comprehensive understanding of the data without employing any coding techniques at this initial stage. After this initial review, codes were inductively created by the lead author (SH) with assistance and frequent review by the second author (NR) in NVivo 12 (QSR International Pty Ltd., 2019) qualitative coding software, We then carefully re-examined the survey responses, assigning additional codes to relevant data that may have been missed during the initial development of the coding matrix. Lastly, we organized the codes into sorted into higher-level categories. The importance of each category was determined based on the number of low-level codes it encompassed. The codes were organized into three hierarchical levels: Level 1 represented the primary categories, Level 2 comprised subcategories derived from these codes, and Level 3 included finer, more detailed meaning units. Table A1 displays the primary categories alongside the Level 2 codes. The complete codebook is available in Supplementary File 1. A sunburst hierarchy chart is presented in Figure A1, which demonstrates the complexity of our coding work. A sunburst diagram is a type of radial tree map used to visualize hierarchical data (QSR International Pty Ltd.). It consists of concentric rings, where each ring represents a different level of the hierarchy. The innermost ring indicates the top level. These rings are further segmented to represent the children or subcategories within each level. This layout is particularly useful for identifying the largest contributing segments or subcategories within a multi-level hierarchy, which in the case of this study, represents caregiver support priorities.

In ensuring the rigor of this qualitative study, we adhered to the principles outlined by Lincoln and Guba (1986) concerning credibility, transferability, dependability, and confirmability. Credibility involves the accurate portrayal of participant realities by the researcher (Lincoln & Guba, 1986). In this study, credibility was supported by continuous communication between the first and second authors (SH and NR) during the analysis of data. Additionally, regular updates were given to the whole research team and a local non-profit Caregiver Committee, facilitated by the Saskatoon Council on Aging and co-chaired by authors LH and SP. Transferability is the extent to which study results can be applied to other contexts, facilitated in this case by reviewing relevant literature (Lincoln & Guba, 1986). Prior to this study, we conducted and subsequently published a scoping review of caregiver support priorities identified by caregivers (Hall et al., 2022), which allowed us to understand the extent of priorities caregivers identify globally. Dependability involves creating an audit trail (Lincoln & Guba, 1986), achieved in this study by maintaining detailed notes on the analysis process and noting any limitations of the study and its data collection methods (Hall et al., 2024b). Finally, confirmability was ensured through salient immersion in the data and by adhering to the content analysis process described by Hsieh and Shannon (2005).

## Results

### Participant characteristics

A total of N = 355 caregivers responded to our online qualitative survey. Out of these, n = 352 participants addressed the query about the obstacles faced by caregivers. The average age of the participants was 61 years (with a range from 22 to 87 years), while the average age of those they cared for was 83 years (ranging between 55 to 104 years). The distribution of participants’ locations was fairly balanced across different settings: large urban areas (33%), small to medium urban areas (32%), and rural areas (35%). The majority of participants were adult children caring for their parents, followed by spousal caregivers. The most frequently reported health condition of the care recipients was dementia, with heart, kidney, lung diseases, and cancer also being common. One aspect not gathered in the survey was the sex or gender of the participants. This was an oversight in our data collection methodology. However, our study did not aim to examine the variances in caregiver experiences and viewpoints based on sex or gender, but rather the overall caregiver experience in Saskatchewan, Canada.

### Qualitative findings

The third survey question asked participants: “What do you think is most important for support in your caregiving role? In other words, what are your top priorities for support?” The overarching categories, which are referred to as the support priorities themselves, were: (1) “help when we need it!”; (2) an ear to listen and a shoulder to lean on; (3) optimizing the care recipient's health; (4) healthcare professionals that care; and (5) improved policies, legislations, and regulations.

#### “Help when we need it!”

“Help when we need it” was the category developed with the largest number of references and is thus the top priority for support shared by participants. Help requested included access to homecare, long-term care homes, information, assistance with daily tasks, and more resources in general. Many participants responded that there are simply not enough services in Saskatchewan to assist them in their caregiving role. Some called for better coordination of the services that do exist. “When I reached out for help and support, I felt like I had to get upset and beg for help and resources to help me with my parent etc.” (Caregiver to a parent with cancer in a small/medium urban setting).

It became clear that access to homecare is something many Saskatchewan caregivers struggle with accessing. Rural participants note that homecare is often only available in urban centres and that “rural people are forgotten.” The costs of homecare were a barrier to receiving help, as many participants reported needing to hire private aides to help them in their caregiving role. Participants also wrote that homecare cannot come visit often enough, leaving much of the work remaining for the caregiver. Lack of consistency in homecare providers leads to caregivers having less trust in homecare services. “Consistency. The home care nurses are seldom the same. They don't know what has happened the previous visit. Every visit feels like starting over with the care.” (Caregiver to a parent with cancer in a small/medium urban setting).

Access to long-term care homes was reported to be essential with Saskatchewan's aging population. Participants recognized that as older adults age with their spouses, ensuring togetherness between spouses in care homes is critical to maintaining their wellbeing. A caregiver to parents with dementia and a heart/kidney/lung condition in a rural setting wrote: “In our case, it was keeping my folks together in the same facility. Whatever level of care was needed for each of them, they were both aware of each other and needed to be together.”

Participants reported feeling disappointed in the care the care recipient received in some facilities, which was related to them feeling as though there was no help available. They called for expansion of services provided in care homes, as well as fair and equitable placement for the care recipient via a smoother admission process. One participant in a large urban setting noted that it took the care recipient 10 months to find placement in a care home, and in the meantime, the caregiver reported having a “breakdown.” Other individuals reported a similar sentiment, noting it was too difficult to qualify for appropriate nursing care. Participants reported wanting to know that the care recipient is safe while in care. Access to this assurance became especially difficult with visitor restrictions during the COVID-19 pandemic, when family caregivers were unable to visit the care recipients in care. As well, some participants reported that there is not enough nursing observation in long-term care homes. They report that they would feel more comfortable with regular checks of vital signs, medication assessments, and mental wellbeing. Caregivers to older adults with dementia wished that staff in care homes had better training on how to provide care to this population.

Participants caring for relatives in the community expressed needing more help with offering daily tasks in their role. These daily caregiving tasks included activities of daily living such as receiving adequate nutrition and personal hygiene care, and instrumental activities of daily living such as grocery shopping, banking, and housekeeping. Other tasks that participants noted needing more help with included blood glucose monitoring, medication administration, wound management, blood pressure management, filling out forms and paperwork, meal preparations, scheduling appointments for multiple specialists, and transportation. “Ensuring that she is well fed, well dressed, and kept clean. After those basics are met, ensuring that she is stimulated and feeling that she is a part of the house.” (Caregiver to a spouse with dementia in a large urban setting).

When caregivers need information, they want to be able to access it freely and easily. Participants noted the lack of a central place or person for information within the province. They called for better signposting and advertisements for resources as well. This navigation assistance and wayfinding for support was mentioned several times throughout responses but participants also wanted to know they could trust the information they were receiving. “Knowing who to ask for help and having access to that resource on a consistent basis as well as having the contact be the same individual over time rather than a difference person every few days. Very frustrating.” (Caregiver to a parent with cancer, frailty, and vision deficits in a large urban setting). In addition to assistance in navigating complex systems, participants wanted to be able to understand what was going on with the care recipient's medical conditions. They want to understand the conditions themselves, but also receive updates on current health statuses, learn how to manage symptoms, and make appropriate decisions for the care recipients.

Rural participants reported sometimes struggling with finding assistance from any service altogether. Rural participants also noted the barrier of transportation in their responses to this survey question, describing needing to travel long distances to receive the help they and the care recipient need. The financial cost of travelling and other rural expenses was also mentioned. A caregiver to a sibling with dementia in a rural setting shared:

Rural support for those who live in rural. I will need to sell my home and move to city as my sister's condition worsens as there is zero support in rural SK. Cost of living in the city is at least three times what it is in the rural.

Respite is crucial to caregivers being able to take care of their own health. Day programs for care recipients are few and far between, but appreciated when they exist. Participants reported sometimes bringing the care recipient to the hospital to be admitted in order to receive some respite for the feelings of burden they may be experiencing. Caregivers call for holistic support for themselves within respite periods, through methods such as yoga classes, massage therapy, and psychotherapeutic counselling. These ideas were brought about by participants; however, other participants felt as though it was hard to step away from their caregiving role when there were not individuals they could fully rely on and trust, to which cost was often a barrier. “Being able to step away when needing a break and have someone responsible to care for your loved one. Home supports that are cost effective.” (Caregiver to a parent with a heart/kidney/lung condition in a large urban setting).

#### An ear to listen and a shoulder to lean on

Feeling heard and understood is critical to caregivers feeling supported, which includes feeling supported by family, friends, social groups, healthcare providers, and their communities. “Having someone you can approach, if there is a problem, and you will not feel judged. There may not be an answer to your problem but often all you need is a listening ear.” (Caregiver to a relative with dementia, a heart/kidney/lung condition, and cancer in a large urban setting). Participants reported valuing when they receive flexibility from those around them and when other individuals take the time to listen to their story. In fact, many participants noted that they often just need someone to listen to their story to feel supported. “A doctor who understands. In [our] case the doctor did not understand. Respite care for the caregiver, and or someone to talk to about the situation.” (Caregiver to a spouse with dementia in a small/medium urban setting).

Another caregiver to a parent with dementia in a rural setting described the idea of being grounded by their social network when they shared that they appreciate when “family and friends … pull you back into the ‘real world.’” Social networks and having individuals to connect with are so important to caregivers, and participants reported that receiving mental health support and having access to support groups is foundational to facilitating their wellbeing while acting as a caregiver. Unfortunately, not all participants had the opportunity to form these connections. “Most important for me was realizing that I could not continue doing it every day - it was affecting my mental health. I wish there would have been support for me.” (Caregiver to a parent with a neurological disorder in a small/medium urban setting). A caregiver to a parent with dementia in a small/medium urban setting stated:

I don’t have enough support. I often feel alone and isolated. Siblings stay away as they want to remember mom as she was. My husband and children allow me to vent my feelings and frustrations to a point. My online dementia group helps me to know I am not alone.

Regarding support groups and networking, participants also reported that they appreciate having access to online support and support via phone. As well, these methods of interaction allowed participants to be more flexible in receiving support. “Online communication or support would be great as I could reach out when I need it without announcing the issues out with her present and allows me to communicate when I can’t leave her alone.” (Caregiver to a parent with dementia in a large urban setting). Bereaved caregivers are sometimes left to deal with guilt and feelings of inadequacy after their active caregiving role comes to an end due to feeling unsupported by the people around them. A caregiver to parents with dementia, cancer, and frailty in a rural setting wrote:

Taking care of [terminally ill] parents causes several mental health care issues where there was no support for the caregiver, regaining life after loss is extremely difficult as no matter what you did, they die anyhow, but you still feel you failed even though you didn't – the disease took them.

Participants who are facing their caregiving duties alone wish they had someone to share the responsibility of their caregiving role with. Some individuals referred to this support as “having backup.” Others noted that care recipients sometimes just need to see a different face other than the primary caregiver, so they called for more companionship from others when the caregiver is away. One caregiver stated they need “someone to take over when needed.” A Caregiver to a parent with dementia in a large urban setting shared:

I want others to share the load of responsibility and assist my mother to have good mental health and not be so alone. She needs more visitors and outings. Mom with some vision loss is insecure about walking. She would benefit from someone taking her out for short walks.

#### Optimizing the care recipient's health

Participants prioritized receiving support in optimizing their care recipients’ health, which involved ensuring the care recipient has their physical and emotional needs met, has their necessities, is mentally stimulated, is physically active, safe, and receiving the best care possible. The participants shared that if the care recipient's health is optimized, it facilitates the caregivers in allowing the care recipient to age in place. Participants wrote that healthcare appointments in Saskatchewan need to be easier to make and happen sooner. They also prioritized themselves having compassion and patience towards the care recipient. Examples of these sentiments in the responses include “Being patient when she asks for help,” “Being emotionally supportive, caring, and compassion[ate],” and “Patience, being strong mentally and not letting him give in.” Lastly, optimizing medication regimes was something participants felt was important to their care recipient receiving the best care.

Ensuring emotional needs are met includes assisting the care recipient in coping with their condition, enhancing connection and preventing isolation for the care recipient, ensuring that the care recipient has a sense of belonging and does not feel burdensome, helping the care recipient maintain independence and dignity, and facilitating the care recipient receiving mental health supports, just as the caregiver needs as well. “I never ever want Mum to think that she is a burden to the family.” (Caregiver to a parent with frailty and arthritis in a rural setting). Ensuring the care recipient is mentally stimulated included responses that highlighted the needs of healthcare staff to help prevent loneliness in the long-term care home. As well, participants reported needing support in strategizing how to prevent loneliness and isolation in the community.

Ensuring the care recipient has their physical needs met encompasses ensuring the care recipient is hygienic and receiving proper nutrition. Many caregivers noted that it is difficult to get the care recipient to maintain proper nutrition, as they report the care recipients having poor appetite and sometimes lack of access to good quality healthy food. Participants made note of how important it is that the caregiver receive information related to the topic of nutrition. Lastly, it was important to participants that the care recipient remains physically active. They need support in keeping the care recipient active through regular outings, walks, and other exercise. Some participants reported the lack of activities for older adults to keep them active. Other caregivers reported the inability to keep themselves active. Throughout many of the responses, it was clear that physical activity also contributes to a healthy consistent routine for both the caregiver and care recipient.

#### Healthcare professionals that care

When participants reported interacting with healthcare professionals that were caring and compassionate, they felt more relieved, relaxed, and less burdened. However, those who had negative experiences with healthcare professionals experienced more subjective strain and hardship. Negative interactions also made it more difficult for participants to act as advocates for the care recipients in their caregiving role. Some participants reported that healthcare professionals in some settings did not seem concerned about what they did or how they provided care, which in turn, led to issues with trust and heightened caregiver anxiety regarding the safety of the care recipient. Importantly, participants want their care recipients to receive more personal and individualized care with compassion, rather than to just receive pills once or twice a day. “We need to be able to trust those assisting our relatives.” (Caregiver to a parent with a heart/kidney/lung condition in a small/medium urban setting). A caregiver to a parent with dementia in a small/medium urban setting wrote:

Having medical staff in both long-term care and hospitals that have the training, time, patience, and stamina to truly provide gentle and effective care - not just hand out pills and stick to their schedules. I know there are many who truly love their patients, but they have no time to let them know it in tangible caring ways.

Participants recognized the value of allied healthcare professionals, such as physical therapists, occupational therapists, social workers, and dietitians; however, they express dismay at how hard it is to access help from these allied health professionals, especially when situated in the community. A caregiver to a parent with dementia, frailty, and mental illness in a large urban setting said:

Adequate physiotherapy, occupational therapy, and home care for senior citizens, so they could have the best possible quality of life and families would not have to provide these services out of pocket. For example, my father is in a public care home where he is only eligible for one physio appointment a month.

Participants reported that poor bedside manner received from healthcare professionals was discouraging and that they want to receive appropriate care, free of judgment. They want to be recognized with the care recipient as a caring dyad and treated as an equal member of the healthcare team. A caregiver to a parent with dementia and a heart/kidney/lung condition in a large urban setting shared:

When my mother was dying in a long-term care facility, I had to go to nursing management to have her care plan include proper positioning, care of bed sores and pain management. My mother was unable to speak and express her needs. Pain meds were often 2–3 h late and I had to find staff to remind them. This was not always met with pleasantness from staff.

Since the caregiver is often the person who spends the most time with the care recipient, they feel as though they know the care recipient's situation well. However, some participants reported not having their insights valued when the care recipient was receiving assessment by healthcare professionals. As such, participants call for enhanced communication between healthcare professionals and themselves and want to be included in the healthcare professional's assessment of the care recipient.

#### Improved policies, legislations, and regulations

The last priority for support was for improved policies, legislations, and regulations to better assist them in their role, which included financial support and support in the workplace for those juggling caregiving and paid employment. A caregiver to a parent with frailty in a large urban setting stated:

There's not enough legislation that allows for long term caregivers [to receive] the understanding and acceptance they need. Familial accommodations in the workplace can be done when young families are having difficulties accessing day cares, etc. When you’re caring for an older adult, the patience or tolerance isn’t the same. There is an unwritten expectation that your parent can be ‘placed in respite’ or ‘admitted to LTC’ when there is conflict between caregiving and working.

Staffing issues in healthcare and long-term care homes were recognized to be a barrier to care recipients receiving adequate care, which in turn placed more strain on the caregivers. As well, there were often small and seemingly insignificant factors that led to care recipients not qualifying for services, such as Meals on Wheels or even long-term care home access altogether. One caregiver to a parent with dementia, a heart/kidney/lung condition, and cancer in a rural setting stated “Very difficult to get support when needed. So many hoops to jump through, only to be told we don't qualify.” This barrier is frustrating for the participants, and once again, more strain is placed on the caregivers.

Participants noted Saskatchewan's lack of intermediate care facilities but also the lack of affordable assistance and care. It was echoed by participants in all geographic settings throughout responses that “caregiving is a full-time job with no pay.” Therefore, it makes it incredibly difficult to afford supportive care when there is limited income received due to the time invested as being a caregiver.

Participants called out the lack of government funding to facilitate caregivers keeping the care recipients in the community to age in place. They also expressed their dismay at the lack of tax breaks and subsidies for caregivers. “SK Government tax breaks for full time caregivers. What is offered now does not even cover one month's expenses - i.e., time off from work and travel to city as rural has no support.” (Caregiver to a parent with dementia in a rural setting). They reported needing more financial assistance to hire relief and also mental health supports for themselves, as many could only access services such as counselling if they had private benefits. “The health care system is too medically, and facility driven and is almost impossible to access unless the patient goes through emergency services.” (Caregiver to a parent with cancer in a rural setting).

When participants had supportive workplaces, their situation was made easier to cope with. On the other hand, when participants did not have supportive employers/woprkplaces, their role was made much more difficult. Participants stated that it is often difficult to take any time off work and those without family supports are the ones who struggle the most with this. Some participants mentioned needing to use up their vacation time to fulfill their role as a caregiver. Many compared caregiving for older adults to parenting children, since parents are more easily afforded time off to provide care to young children, but the standard is not set to be the same with caregivers to older adults. “It is important that employers allow special days for times she needed me as I was the only family member nearby to help her. This is a societal issue…” (Caregiver to a parent with dementia and post-stroke in a rural setting).

## Discussion

This study reveals the multifaceted support priorities of caregivers of older adults in Saskatchewan, Canada. Their primary needs encompass not just practical assistance, but also emotional support, professional healthcare engagement, and systemic changes in policies and regulations. These insights emphasize the necessity for a holistic approach in supporting caregivers, recognizing their critical role in and contributions to the healthcare system. The most emphasized need was for immediate, tangible help in the form of homecare, access to long-term care homes, information, and assistance with daily tasks. Many caregivers expressed frustration with the scarcity and inadequacy of available services, especially in rural areas. The high cost and lack of consistent homecare services further compounded these challenges.

With little access to homecare, it is an increasingly regular expectation that caregivers acquire a sophisticated understanding of their care recipient's condition, as well as new skills to execute complex medical or nursing tasks (Bell et al., 2019). Despite these pressing responsibilities, caregivers are also expected to complete these tasks without adequate preparation and support (Bell et al., 2019). Caregivers who report lack of care coordination, respite breaks, continuity of care, and difficulties with prioritizing their own needs have been found to have poorer self-care behaviours (Oliveira, Zarit, et al., 2019b). In a community assessment of caregivers in Saskatoon, Saskatchewan (Hall & Holtslander, 2022), caregivers reported they need better access to respite services, which was echoed by participants across the province in this study. The mental health effects of providing respite to caregivers are numerous, including an improvement in life satisfaction, overall wellbeing, and a decrease in self-reported depressive symptoms (Costa-Font & Vilaplana-Prieto, 2022).

Some participants in our study were caregivers of older adults in care homes, which we allowed for in our inclusion criteria (caregivers providing care at any level of involvement to older adults). As such, it is worthwhile to note that caregivers of older adults who reside in care homes face different challenges and may have different support needs compared to those providing care in the home (Davies & Nolan, 2006). Previous research shows that caregiving responsibilities, although differing in context, often persist with a similar intensity even after a care recipient moves into a care facility (Keefe & Fancey, 2000). Overseeing the care of relatives in long term care reflects a continuity of care rather than a cessation (Keefe & Fancey, 2000). Caregivers shift from direct care tasks to advocacy and coordination roles, which are crucial for ensuring the well-being of care recipients in these settings (Keefe & Fancey, 2000). Recent dialogues in caregiving research advocate for recognizing the dynamic and adaptable nature of caregiving across different care contexts (Harvath et al., 2020). The adaptation of caregiving roles from direct care in community settings to advocacy and oversight in care homes reflects a broader continuum of caregiver involvement, underscoring the need to consider all aspects of caregiving in studies which aim to understand the caregiver experience (Harvath et al., 2020).

Participants in this study noted the physical strain of the caregiving role. As noted in other studies, caregivers can have their own physical limitations that make providing care difficult (Oliveira, Vass, et al., 2019a; Oliveira, Zarit, et al., 2019b). Anxiety accompanies this physical strain, as caregivers sometimes worry about the fact that their own health issues may eventually prevent them from caring for their care recipient (Oliveira, Vass, et al., 2019a). With one in four older Canadians acting as caregivers to their spouses (Arriagada, 2020), it is imperative to ensure sufficient help exists as the physical aspects of caregiving become more demanding, which also holds true for younger caregivers (Koumoutzis et al., 2021). Transportation is a frequently reported barrier to care, which necessitates the involvement of caregivers in providing this service (Bell et al., 2019). For those in rural settings, participants in this study reported that transportation is even more complicated due to the extended distance needed to travel to appointments. In a study of barriers and facilitators to stroke recovery (Magwood et al., 2019), community ride-sharing programs have been found to be facilitators that mitigate transportation being a barrier.

Support from social networks and healthcare professionals is a key facilitator to caregivers’ self-reported life balance (Brooks et al., 2022; Rossau et al., 2022; Tokovska et al., 2022). Caregivers have previously reported how caregiving limits their social lives and brings on psychological strain (Rossau et al., 2022). When psychosocial support is provided to caregivers in a comprehensive and systematic way, caregivers are empowered to handle the emotional, cognitive, and behavioural consequences of their role (Tokovska et al., 2022). Caregivers to persons living with dementia widely endorsed the value of support groups, which offered companionship as well as information, noting the opportunity to meet others who were experiencing similar situations is therapeutic (Toms et al., 2015). There is also stress, depression, loneliness, guilt, and grief when caregivers reach a point where they need to place their care recipient in care (Brooks et al., 2022). Brooks et al. (2022) reported that psychoeducational and psychosocial supports help caregivers to better cope with the stressors and losses experienced during the transition from caregiving at home to caregiving in the periphery while care recipients enter professional care settings. As these findings from previous studies align with those in this study, that psychoeducational and psychosocial supports should be amplified where existing, and made available where absent within Saskatchewan.

Caregivers often engage in protective behaviours to safeguard their care recipient's physical and emotional wellbeing, which can result in detrimental effects on the caregiver (Jeyathevan et al., 2019). Caregivers play a crucial role in optimizing their care recipient's health and well-being. Caregivers act as advocates, share in decision-making, and also provide basic care such as feeding, hygiene, and socialization (Nash et al., 2021). Regarding optimizing the emotional health of care recipients, caregivers to individuals with spinal cord injuries noted that they hide their distress related to caregiving from care recipients to protect the care recipient from feeling guilty (Jeyathevan et al., 2019). In their study of cancer caregivers, Sun et al. (2019) called for interventions that provide personalized support to caregivers in meeting the physical and emotional demands of the care recipient's cancer. Most caregivers strive to provide high-quality care (Meyer et al., 2022). It is clear from the findings within this study and in the wider body of literature that caregivers need more support in ensuring the care recipient's health conditions are optimized. When health conditions are not optimized, caregivers report strain in relationships and higher levels of stress related to their role (Meyer et al., 2022).

Situating this study's findings within the wider literature can help to assist healthcare professionals in their provision of effective and inclusive care. Caregivers often feel unprepared for their caregiving role and consequently require additional support from healthcare professionals for the practical aspects of caring and general information regarding the care recipient's conditions (Becqué et al., 2019; Cochrane et al., 2022). Caregivers often act as primary information seekers and need to be able to act on the information retrieved to be able to better understand and manage the care recipient's conditions (Cochrane et al., 2022). As well, to reduce feelings of uncertainty, caregivers report wanting information regarding the care recipient's prognosis and life expectancy when facing life-limiting illnesses (Preisler et al., 2019). Appointments with healthcare professionals are an opportune time to conduct this gathering of information, which highlights the importance of having a caregiver present during appointments (Cochrane et al., 2022). A finding from the present Saskatchewan study was that participants prioritize respectful and helpful bedside manner from the healthcare professionals they interact with and want to feel included during the care recipient's appointments. Caregivers in the study by Cochrane et al. (2022) viewed interactions with healthcare professionals as their main opportunity for accessing information, although when they felt uncomfortable with the healthcare professional, they were more reluctant to ask questions.

### Canadian context

Canada's long-term care system has been criticized in policy for needing an ‘overhaul’ (Flood et al., 2021). Challenges that Canadian policy-makers face involve not only adequately meeting the growing needs for long term care services, but also ensuring that those services are delivered where people want to receive them, which is most often at home (Flood et al., 2021). *Cash-for-care* benefits are direct public transfers paid to caregivers to support care at home, and are widely implemented in countries such as Germany and the Netherlands (Flood et al., 2021). However, as of 2021, Nova Scotia is the only Canadian province to implement a cash-for-care caregiver benefit program, in which caregivers receive a sum of $400 per month (Mihailescu, 2021). Although Nova Scotia's aging population is parallel to that of Saskatchewan's, Nova Scotia took the initiative to implement the *Continuing Care Strategy*, setting the stage for implementation of more caregiver supports (Mihailescu, 2021). Access to this benefit allows care recipients to remain in their homes to age in place for a longer amount of time, which was anticipated to decrease healthcare spending overall (Mihailescu, 2021). O'Hara (2014) compared the caregiver benefit program in Nova Scotia with the Manitoba Caregiver Tax Credit through secondary data analysis and interviews with policy experts. It was reported that clients receiving the benefit in Nova Scotia are 56% less likely to be admitted to long term care facilities (O'Hara, 2014). Therefore, Saskatchewan's current systems should focus on positive aging in the community, in addition to supporting those in care. Our knowledge translation efforts for the Saskatchewan Caregiver Experience Study include presenting our work to stakeholders and healthcare professionals at a national conference on geriatrics and gerontology (Hall et al., 2023), as well as approaching Saskatchewan's provincial and federal politicians with our findings and suggestions.

### Global context

These global studies we discussed demonstrate that the findings from Saskatchewan are consistent with the experiences of caregivers in different parts of the world, highlighting common challenges and needs. The transferability of these findings to more settings is evident in the shared themes of required support, whether in terms of practical assistance, emotional support, or policy changes. This finding suggests that the insights gained from the Saskatchewan study could inform caregiving support strategies in various global contexts.

The challenges faced by caregivers in Saskatchewan, Canada, are mirrored globally, with unique nuances in different regions. The global aging population is leading to a higher demand for caregivers. This demand is particularly evident in countries with aging populations where managing chronic non-communicable diseases and neurodegenerative diseases like dementia is becoming a significant challenge (Fang et al., 2020). Caregivers worldwide have identified similar priorities, including orientation to the caregiving role, self-care and respite, adapting healthcare, improved supports, information needs, access to resources, and financial assistance (Hall et al., 2022). These findings align with the Saskatchewan caregivers’ emphasis on access to homecare, long-term care homes, information, and assistance with daily tasks. Research indicates considerable variability in caregiving situations based on the health condition of the care recipient, regional differences (urban vs. rural), and cultural contexts. This diversity necessitates tailored interventions and support systems (Young et al., 2020). Furthermore, the need for emotional and psychosocial support, such as a listening ear and someone to lean on, is echoed in global studies. The 2021 Alzheimer's Disease and Related Dementias National Consensus Conference recommended enhancing caregiver engagement and support in healthcare settings through identification, assessment, reimbursement, training, education, and technology (Riffin et al., 2022). These forms of support are crucial for caregivers’ mental health and well-being (Riffin et al., 2022).

Caregivers globally have emphasized the importance of healthcare professionals who are caring, compassionate, and considerate of the caregiver's role and insights (Muñoz-Bermejo et al., 2020), similar to this Saskatchewan-based study. Moreover, caregivers across various regions have called for improved policies, legislation, and regulations to better support them, highlighting the need for financial assistance and workplace accommodations (Petry et al., 2022), similar to the needs expressed in Saskatchewan. Direct compensation, and regular respite care can help support caregivers in their role (Petry et al., 2022).

### Strengths and limitations

A strength of our study was our qualitative descriptive approach (Sandelowski, 2000, 2010), as it provided a straightforward description of the data, organized by topic and relevance. This approach was particularly suitable for the Saskatchewan Caregiver Experience Study, as we aimed to identify caregivers’ priorities for support. Despite being one of the least theoretical approaches in qualitative research, qualitative description is ideal when describing a phenomenon is the main goal (Neergaard et al., 2009). The online data collection format made participation more accessible for caregivers, eliminating the need for arranging care for their recipients. This method aligns with the growing trend of utilizing online platforms for qualitative research (Carter et al., 2021; Horrell et al., 2015).

Limitations also exist in this study. The study did not collect data on caregiver gender. This was a significant oversight, especially considering the importance of understanding diverse caregiver experiences. However, as previously stated, our aim was not to describe gender-based differences in experience, but rather the overall experience of caregiving in Saskatchewan. As well, the study's participant pool was formed through voluntary self-selection, which might have introduced a bias based on the self-selection process. This self-selection method means that those who opted to join the study could potentially have stronger or more specific opinions, which might influence the overall results. The study also faced a potential limitation in its representation of caregivers who may not be adept with technology or lack access to digital platforms. The reliance on an online survey method may have inadvertently excluded individuals who either do not have internet access or are not comfortable with utilizing digital platforms, thereby possibly omitting certain demographic groups more unfamiliar with such methods.

## Conclusion

The Saskatchewan Caregiver Experience Study maps the complex and multifaceted perspectives of caregivers for older adults in Saskatchewan, Canada. Our findings underscore the pressing need for a comprehensive and nuanced approach to caregiver support, addressing both practical and emotional aspects. Caregivers clearly articulate their need for practical assistance in terms of homecare, long-term care, and help with daily tasks. This need is particularly acute in rural areas, where resources are scarce and accessibility is limited. Emotional support, including a listening ear and a shoulder to lean on, is also vital, highlighting the importance of social networks and mental health support for caregivers. Professional healthcare engagement emerged as an important factor, with caregivers emphasizing the need for healthcare professionals who are caring, compassionate, and inclusive of the caregiver's role and insights. Systemic changes in policies and legislations are urgently required to address the challenges faced by caregivers. This includes financial support, workplace accommodations, and more inclusive and equitable healthcare policies. Optimizing the care recipient's health is another priority, with caregivers striving to ensure that their care recipients receive the best possible care, both physically and emotionally.

This study extends its relevance to a global audience, providing insights into the support priorities of caregivers, which have been previously reported (Hall et al., 2022), but not in geographic contexts such as ours, with an even distribution among urban and rural settings. Our findings highlight themes in caregiver needs across urban and rural geographic contexts, emphasizing the importance of accessible support, the role of healthcare professionals, and the impact of policies on caregiver efficacy. These insights can inform caregiver support strategies both nationally and internationally, making a contribution to the understanding of how best to support caregivers in diversely distributed settings. The study also offers critical insights for policymakers, healthcare providers, and community organizations. It calls for a holistic approach to caregiver support, recognizing the indispensable role of caregivers in the healthcare system and the diverse challenges they face. As the population continues to age, it is imperative that health and social systems respond to the needs of caregivers with empathy, respect, and action, ensuring that they receive the support they need to continue their invaluable work.

## Supplemental Material

sj-docx-1-cjn-10.1177_08445621241273956 - Supplemental material for The Saskatchewan Caregiver Experience Study: Support Priorities of Caregivers of Older Adults Supplemental material, sj-docx-1-cjn-10.1177_08445621241273956 for The Saskatchewan Caregiver Experience Study: Support Priorities of Caregivers of Older Adults by Steven Hall, Noelle Rohatinsky, Lorraine Holtslander and Shelley Peacock in Canadian Journal of Nursing Research

## Appendix

**Table A1.** Overarching categories and subcategory (Level 2) codes.

Overarching category	Subcategory codes (Level 2)
“Help when we need it!”	Access to home care Access to long term care Information when we need it! More resources are needed for caregivers Assistance with daily tasks
An ear to listen and a shoulder to lean on	Respite for caregivers Mental health support for the caregiver Feeling heard and understood Networking with other caregivers Online support Grounding yourself Reassurance and validation Support via phone
Optimizing the care recipient's health	Ensuring the care recipient is safe Ensuring the care recipient has their emotional needs met Ensuring the care recipient is mentally stimulated Ensuring the care recipient has their physical needs met A consistent routine Ensuring the care recipient has the best care Ensuring the care recipient is physically active Knowing their care recipient is safe in their own care Aging in place Ensuring the care recipient has the necessities
Healthcare professionals that care	Better communication with the caregiver Access to allied health professionals More health assessments done on care recipients More HCPs to help caregivers Better healthcare in rural and remote areas Receiving care with dignity and respect
Improved policies, legislations, and regulations	Financial support Appropriate staffing in hospitals and care homes Supportive workplaces A proper drivers’ assessment for the care recipient More help for the little guy who doesn’t qualify
