# Supplementary material for: The Saskatchewan Caregiver Experience Study: Support Priorities of Caregivers of Older Adults
Source: Can J Nurs Res. 2024 Aug 11;56(4):418–31. doi: 10.1177/08445621241273956 (PMC11528855; doi:10.1177/08445621241273956)
Supplement: sj-docx-1-cjn-10.1177_08445621241273956 - Supplemental material for The Saskatchewan Caregiver Experience Study: Support Priorities of Caregivers of Older Adults [file sj-docx-1-cjn-10.1177_08445621241273956.docx]

Saskatchewan Caregiver Experience Study

Question 3

*“What do you think is most important for support in your caregiving role? In other words, what are your top priorities for support?”*

## Nodes (coding aggregated from child nodes)

| Name | References |
| --- | --- |
| **(1) Help when we need it** | **62** |
| Access to home care | 33 |
| Access to hospice care | 1 |
| Home care visits | 7 |
| Access to long term care | 4 |
| Ensuring togetherness between spouses in care homes | 2 |
| Expansion of services provided in care homes | 1 |
| Fair and equitable placement in care | 2 |
| Intermediate care facilities | 1 |
| Knowing their care recipient is safe in their care home | 2 |
| More nursing observation in long term care | 1 |
| Palliative care | 2 |
| Quality LTC | 4 |
| Assistance with daily tasks | 1 |
| Access to mobile phlebotomy | 2 |
| Assistance with blood glucose monitoring | 2 |
| Assistance with medication administration | 16 |
| Assistance with the care recipient's ADLs | 7 |
| Assistance with the care recipient's IADLs | 7 |
| Caregiver being granted access to pay bills and taxes | 1 |
| Assistance with wound management | 1 |
| Blood pressure management | 3 |
| Daily care assistance available | 5 |
| Grocery delivery | 2 |
| Help with chores and housekeeping | 24 |
| Help with forms and paperwork | 1 |
| Meal prep | 6 |
| Night time help | 3 |
| Pharmacy delivering medications | 1 |
| Physical support | 6 |
| Scheduling assistance | 1 |
| Support with transportation | 13 |
| Information when we need it! | 4 |
| A central place or person for information | 9 |
| Better sign-posting and advertisement for supports | 1 |
| Decision-making assistance | 3 |
| eHealth | 1 |
| Information on complementary and alternative therapies | 1 |
| Knowledgeable advice | 10 |
| Navigation assistance | 9 |
| Knowing where to go for information | 5 |
| Wayfinding for support | 10 |
| Orientation to role | 4 |
| Advanced care planning help | 2 |
| Education on how to provide care needs | 10 |
| Managing symptoms | 1 |
| Pain management | 1 |
| Symptom management at end of life | 2 |
| Feedback for caregivers | 1 |
| Understanding medical information | 5 |
| What are the next steps | 1 |
| Support in bereavement | 1 |
| Telehealth | 2 |
| More resources are needed for caregivers | 3 |
| Affordable help | 12 |
| Rural residents lacking access to healthcare | 10 |
| Transportation as a barrier | 7 |
| **(2) An ear to listen and a shoulder to lean on** | **107** |
| Feeling heard and understood | 12 |
| Feeling supported by family | 27 |
| Feeling supported by friends and social groups | 8 |
| Feeling supported by HCPs | 25 |
| Feeling supported by the community | 1 |
| Grounding yourself | 1 |
| Mental health support for the caregiver | 20 |
| Emotional support | 8 |
| Help finding balance | 1 |
| Networking with other caregivers | 5 |
| Access to support groups | 7 |
| Online support | 2 |
| Reassurance and validation | 1 |
| Respite for caregivers | 55 |
| Being able to take care of their own health | 2 |
| Day programs for the care recipient | 9 |
| Disability programs | 1 |
| Extended periods of respite | 7 |
| Government funding for respite services | 1 |
| Holistic support for caregivers (yoga, massage, counselling) | 6 |
| Knowing their care recipient is safe during respite | 4 |
| Personal time for the caregiver | 2 |
| Respite care closer to home | 2 |
| Someone to be there when the caregiver can't be | 1 |
| A voice for the voiceless | 1 |
| Backup for caregivers | 1 |
| Companionship when the caregiver is away | 1 |
| Support when the primary caregiver is not there | 2 |
| Someone to share the responsibility with | 5 |
| Volunteer respite programs | 1 |
| Support via phone | 1 |
| **(3) Optimizing the care recipient's health** | **66** |
| A consistent routine | 7 |
| Exercise & walking | 9 |
| Aging in place | 1 |
| Facilitating the care recipient staying in their home | 7 |
| Support in supporting the care recipient to stay at home | 1 |
| Ensuring the care recipient has the best care | 5 |
| Appointments happening sooner | 6 |
| Compassion | 3 |
| Optimizing medication regimes | 2 |
| Patience | 6 |
| Ensuring the care recipient has the necessities | 1 |
| Ensuring the care recipient has their emotional needs met | 10 |
| Assistance in helping the care recipient cope | 2 |
| Enhancing connection and preventing isolation | 2 |
| Ensuring the care recipient does not feel burdensome | 1 |
| Ensuring the care recipient has a sense of belonging | 1 |
| Ensuring the care recipient maintains their independence | 5 |
| Extra visits for the care recipient | 7 |
| Maintaining the care recipient's dignity | 6 |
| Mental health support for the care recipient | 4 |
| Ensuring the care recipient has their physical needs met | 8 |
| Ensuring the care recipient is hygienic | 6 |
| Ensuring the care recipient is receiving proper nutrition | 23 |
| Ensuring the care recipient is mentally stimulated | 10 |
| Activities for the care recipient to participate in | 12 |
| Ensuring the care recipient is physically active | 2 |
| Ensuring the care recipient is safe | 14 |
| Knowing their care recipient is safe in their own care | 2 |
| **(4) Healthcare professionals that care** | **34** |
| Access to allied health professionals (PT, OT, etc.) | 7 |
| Better communication between services | 5 |
| Better communication to the caregiver | 8 |
| Being accepted as an advocate for the care recipient | 6 |
| HCPs include caregiver perspective in assessment | 4 |
| Regular follow-up with HCPs | 2 |
| Better healthcare in rural and remote areas | 2 |
| For help to exist in rural and remote areas | 1 |
| Rural and needing to relocate | 2 |
| More HCPs to help caregivers | 3 |
| Training for HCPs to work with caregivers | 4 |
| More health assessments done on care recipients | 4 |
| Receiving care with dignity and respect | 2 |
| Appropriate HCP bedside manner with the caring dyad | 6 |
| Care free of judgment | 2 |
| Caring dyad seen as part of the HCP team | 3 |
| Dementia friendly care | 3 |
| HCPs value the care recipient regardless of their age and diagnosis | 2 |
| **(5) Improved policies, legislations, and regulations** | **44** |
| A proper drivers' assessment for the care recipient | 2 |
| Appropriate staffing in hospitals and care homes | 6 |
| Financial support | 25 |
| Caregiving is a full time job with no pay | 2 |
| Financial assistance while on leave from work | 4 |
| Government funding for caregivers to hire services | 4 |
| Support from insurance programs | 1 |
| Tax breaks for caregivers | 6 |
| Travel costs covered | 1 |
| More help for the little guy who doesn't qualify | 2 |
| Supportive workplaces | 6 |
| Easier for caregiver to take time off work | 7 |
